# Visual/Photoelectrochemical Off-On Sensor Based on Cu/Mn Double-Doped CeO_2_ and Branched Sheet Embedded Cu_2_O/CuO Nanocubes

**DOI:** 10.3390/bios13020227

**Published:** 2023-02-04

**Authors:** Huihui Shi, Yanfei Che, Yumeng Rong, Jiajun Wang, Yanhu Wang, Jinghua Yu, Yan Zhang

**Affiliations:** 1 School of Chemistry and Chemical Engineering, University of Jinan, Jinan 250022, China; 2 Shandong Analysis and Test Center, Qilu University of Technology (Shandong Academy of Sciences), Jinan 250014, China; 3 Key Laboratory of Optic-Electric Sensing and Analytical Chemistry for Life Science, MOE, Qingdao University of Science and Technology, Qingdao 266042, China

**Keywords:** photoelectrochemical, colorimetric, thrombin, paper-based device, Cu_2_O/CuO, CuMn@CeO_2_

## Abstract

An integrated dual-signal bioassay was devised to fulfil thrombin (TB) ultrasensitive detection by integrating visualization with the photoelectrochemical technique based on G-quadruplex/hemin. During the process, branched sheet embedded copper-based oxides prepared with illumination and alkaline condition play a vital role in obtaining the desirable photocurrent. The switchover of photoelectrochemical signal was realized by the adjustable distance between electron acceptor G-quadruplex/hemin and interface materials due to dissociation of the Cu/Mn double-doped cerium dioxide (CuMn@CeO_2_)/DNA caused by the addition of TB. Then, CuMn@CeO_2_ transferred onto visual zones triggered catalytic reactions under the existence of 3,3′,5,5′-tetramethylbenzidine and hydrogen peroxide, making a variation in color recognized by the naked eye and providing visual prediction. Under optimized conditions, this bioassay protocol demonstrated wide linear ranges (0.0001–50 nM), high selectivity, stability, and reproducibility. More importantly, the proposed visual/photoelectrochemical transduction mechanism platform exhibits a lower background signal and more reliable detection results, which also offers an effective way for detecting other proteins and nucleic acids.

## 1. Introduction

Thrombin (TB), as a member of the serine protease group, has been identified to be expressed in various cardiovascular diseases including cerebral ischemia and infarction. Considering the importance of TB assays, an efficient sensing strategy for fast and sensitive TB detection is highly desired [1,2]. Up to now, numerous analytical methods were reported, including chemiluminescence [3], electrochemiluminescence [4,5], fluorescence [6], electrochemistry [7,8], and photoelectrochemistry (PEC) [9,10]. Among these protocols, PEC, as an emerged analytical method with high sensitivity and a low background signal, has received substantial attention [11,12]. However, most of them relied on a signal output model, which still need to be improved to decrease the influence of systematic error. To achieve a more accurate and reliable analysis, an alternative method is to combine a naked-eye colorimetric strategy with the PEC transduction mechanism.

As an essential portion in colorimetric reaction and the PEC process, nanomaterials with catalytic and photoactive activity have a great impact on the obtained signal strength [13,14,15,16]. Cerium oxide (CeO_2_), simultaneously existing Ce^3+^ and Ce^4+^ oxidation states on the lattice surface, could catalyze 3,3′,5,5′-tetramethylbenzidine (TMB) to convert colorless to blue with assistance of H_2_O_2_ due to oxygen vacancy [17,18]. However, the capacity is relatively low to be used for low-concentration analytes detection. It is a good method to dope CeO_2_ with metal to expand their application as colorimetric reaction probes [19,20]. As a consequence, double-doped CeO_2_ (CuMn@CeO_2_) was prepared with superior catalytic performance for more accurate detection. Furthermore, to obtain a desirable PEC signal, a cuprous oxide–cupric oxide (Cu_2_O/CuO) composite was introduced as a photoactive material owing to its abundance, affordable price, environmental acceptability, and low band-gap energy [21,22]. The fabrication process was carried out under normal temperature and pressure, with low-cost and high-quality products. The introduction of an external stimulus can promote the reduction process significantly, and the obtained Cu_2_O in such a strong redox photochemical surrounding possessed the intrinsic superiority to endure photocorrosion in a kinetically more favorable way [23]. Furthermore, the well-designed overall architecture of the branched sheet embedded nanocubic Cu_2_O/CuO complex not only provides more active sites and a larger surface area, but also helps Cu_2_O to overcome its high carrier recombination caused by short electron diffusion length (20–100 nm) [24,25].

Apart from desirable initial PEC performance, an efficient electron transfer-regulated strategy is also of vital importance in broadening the application range of the PEC system [9,26]. G-quadruplex/hemin, which forms between single-stranded guanine-rich aptamer and hemin, has been utilized to act as an electron acceptor and mediate the catalytic reduction of dissolved oxygen [9,27]. Thus, this special structure could be employed as an effective way to realize off-on transformation in the PEC detecting process. In addition, to operate the whole process, an easy-to-operate platform was necessary. Cellulose paper, owning virtues of unique structure, low cost, large specific surface area, and portability, has aroused widespread interest [28,29,30]. Concretely, it is easy to be folded to satisfy diverse demand when applied as micro-reactors. Combined with the abovementioned factors, an integrated paper-based platform was fabricated to functionalize the working electrode and implement a multi-module microfluidic device.

**Scheme 1:**
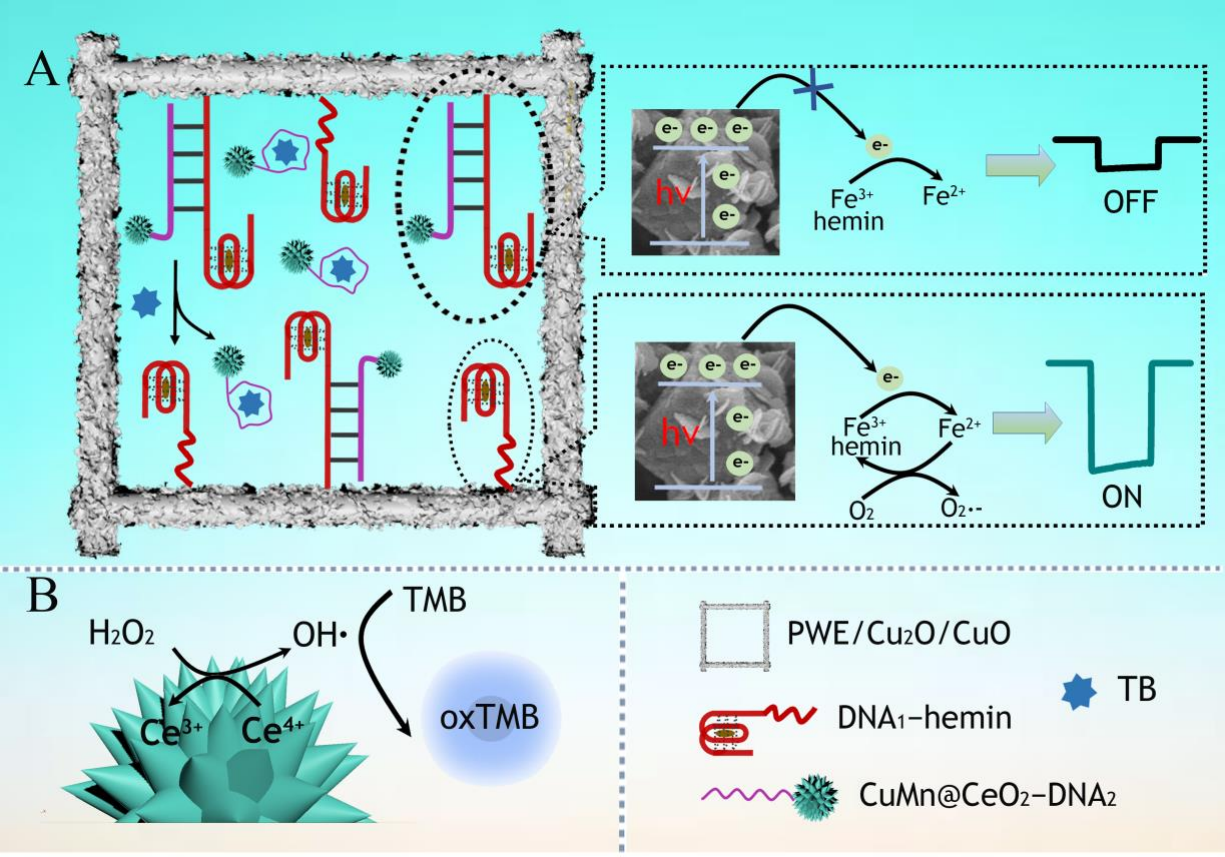
Schematic illustration of (**A**) off−on switchover of PEC areas and (**B**) visual detection of TB.

Hence, a dual-signal output paper-based sensing strategy integrating the PEC technique with visualization in two spatially separated working areas, gold nanoparticles (AuNPs)-modified PEC working electrodes (PWE) and visual areas, was proposed for TB detection (Scheme 1). Concretely, TMB and H_2_O_2_ were applied on the visual areas for chromogenic reactions, while branched sheet-like nanocubic Cu_2_O/CuO and hemin-DNA_1_ were applied on the PWE surface with excellent PEC performance due to G-quadruplex/hemin. With addition of Cu/Mn-doped cerium dioxide/DNA_2_ (CuMn@CeO_2_-DNA_2_), the specific double-stranded structure based on hybridization of DNA_1_ and DNA_2_ and steric hindrance of CuMn@CeO_2_ could dramatically decrease the photocurrent, thus the “signal-off” trend for PEC signal can be triggered. After that, with the application of TB, DNA_2_ could hybridize with TB, causing free CuMn@CeO_2_-DNA_2_. Then, the released CuMn@CeO_2_ could catalyze H_2_O_2_ on visual areas, forming the hydroxyl radical that can make TMB effectively develop colors, which offers visual prediction for thrombin concentration. Meanwhile, due to a DNA_2_-TB binding event, dissociation of CuMn@CeO_2_ facilitated the electrons transfer and G-quadruplex/hemin could accept electrons from the illuminated Cu_2_O/CuO, resulting in the increase of PEC intensity and switchover from off to on mode of the PEC signal. Such a distance-based PEC sensing system between electron acceptor and interface materials endows detection methods with lower background signal. Accordingly, the paper-based analytical device with a dual-signal output sensing mechanism was successfully fabricated, which could be introduced as quantitative platform for screening of other proteins and nucleic acids.

## 2. Experimental Section

### 2.1. Design of Paper-Based Device

**Figure 1:**
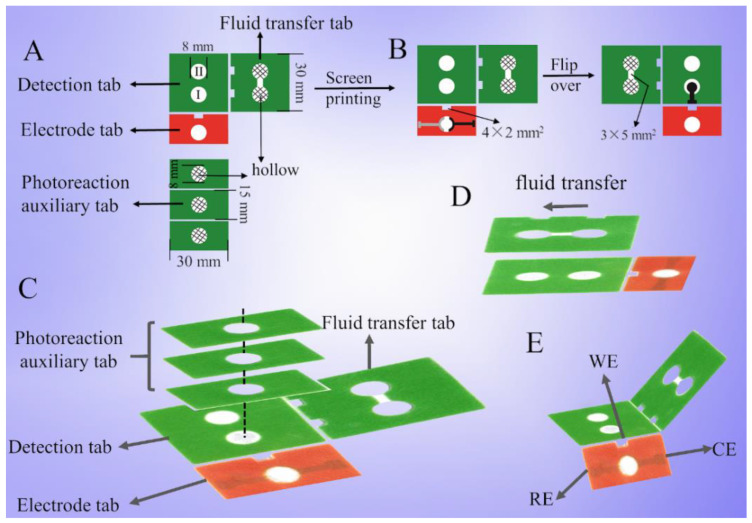
Schematic layout of (**A**) prepared device and (**B**) the fabricated device with screen−printed electrodes. Assembly illustration of prepared device during (**C**) photoreaction, (**D**) fluid transfer, and (**E**) sensing platform.

The prepared whole device was made with four units: detection tab, photoreaction auxiliary tab, fluid transfer tab, and electrode tab, as depicted in Figure 1A,B. Two working zones (8 mm in diameter) for the establishment of the sensing platform were patterned on the detection tab (I for PEC system, II for chromogenic reaction). In order to form a densely distributed nanosized oxide layer, more growth solution that exceeded the capacity of a single working area was required. Thus, the photoreaction auxiliary tab, where the white area was designed to be hollow while the green area is hydrophobic, was added to the device to hold more solutions in the light reaction process after folding (Figure 1C). When the device was folded as shown in Figure 1D, the branch channel (3.0 mm × 5.0 mm) on the fluid transfer tab is favorable to connect two working zones which are ready for fluid transfer. The carbon working electrode (WE) was printed on the back of the detection zone, while the carbon counter electrode (CE) and Ag/AgCl reference electrode (RE) are on the hydrophilic zone (8 mm in diameter) of the electrode tab. Finally, a three-dimensional device for visual and PEC detection was successfully constructed after the electrode tab folded under the detection tab, as displayed in Figure 1E.

### 2.2. Preparation of CuMn@CeO_2_-DNA_2_

Firstly, 0.1 g CuMn@CeO_2_ (the synthesis process can be found in the Supporting Information) was redispersed in 10 mL ethanol and stirred for 30 min. Then, 0.1 mL 3-aminopropyltriethoxysilane was injected into the above solution and refluxed at 70 °C for 90 min. After the obtained solution was centrifugated and washed with ethanol and deionized water, 100 μL glutaraldehyde (1%, *w*/*w*) was added to activate the amino groups on the CuMn@CeO_2_ surface. To obtain CuMn@CeO_2_-modified DNA_2_, 15 µL of DNA_2_ was first added in the dispersed 2 mL CuMn@ CeO_2_ solution with stirring for 12 h at 4 °C. Next, the above solution was diluted with 25 mM Tris-HCl and 0.3 M NaCl, followed by centrifugation at 12,000 rpm for 10 min to remove unconjugated DNA. The resultant CuMn@CeO_2_-DNA_2_ was stored at 4 °C when not in use.

### 2.3. Fabrication of Dual-Signal Sensing Platform

PWE were prepared as shown in the Supporting Information. In order to achieve a desirable PEC signal, nanocubic Cu_2_O was first coated on PWE by the light-induced photochemical synthesis approach [23]. Briefly, 1 mL as-prepared copper tungstate (CuWO_4_) solution (0.15 M, the preparation process can be found in the Supporting Information) was coated onto PWE by a spin coater, then 50 μL solution containing 0.3 M NaOH and 0.1 M glucose was dropped onto the surface of PWE with a photoreaction auxiliary unit. Next, the assembled photochemical reaction device was irradiated by a 300W Xe lamp for 60 min and then rinsed with distilled water. After that, the electrode dealt with 50 μL 0.2 M NaOH for 30 min. Subsequently, 50 μL chitosan aqueous solution (0.08 wt%) in 1% acetic acid was applied onto the working electrode and reacted for 60 min. After drying, 5 wt% glutaraldehyde was added to activate the electrode, and the final electrode was denoted as PWE/Cu_2_O/CuO. Finally, 20 μL acetic acid (pH 4.5), 20 mM TMB, and 20 μL of 0.5 M H_2_O_2_ were dropped onto the II visual area.

### 2.4. Analysis Process of TB

The dual-signal mechanism detection was performed by the following procedure. Firstly, 50 μL of 1 μM DNA_1_ and 1 μM hemin was dropped onto the PWE and kept for 80 min, followed by adding 50 μL of 1 μM CuMn@CeO_2_-DNA_2_. After the PEC electrode was incubated with different concentrations of TB for 50 min, the device was folded as depicted in Figure 1D and the above mixed solution’s fluid flow from PWE to the II visual zone could be generated along the hydrophilic channel on the fluid transfer tab. The electrode was carefully cleaned with 0.01 M phosphate buffer saline (PBS, pH 7.4) after each step. Finally, color variance could be signaled and the PEC signal was recorded by a three-electrode system (−0.1 V).

## 3. Results and Discussion

### 3.1. Structure Characterization

**Figure 2:**
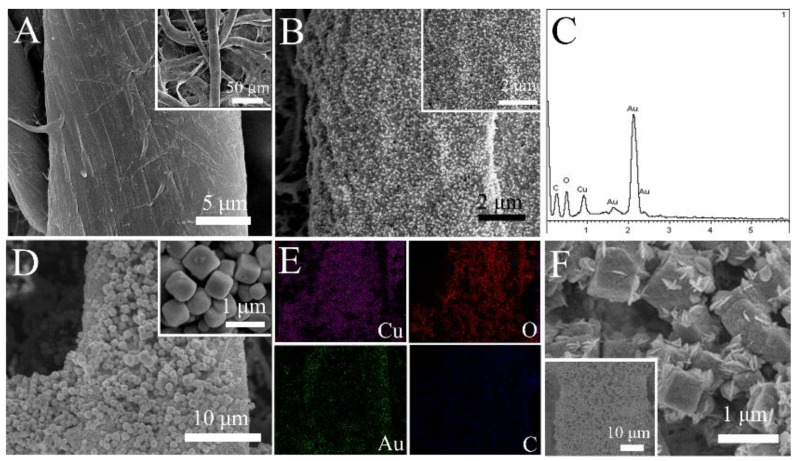
SEM images of (**A**) sample zone, (**B**) PWE, (**D**) PWE/Cu_2_O, and (**F**) PWE/Cu_2_O/CuO at different magnifications. (**C**) EDS and (**E**) elemental mappings of PWE/Cu_2_O.

The morphology evolution of nanomaterials was investigated by scanning electron microscope (SEM) as illustrated in Figure 2. Clearly, it could be found that interconnected fibers of the bare sample zone displayed porous architecture with high surface area (Figure 2A), which provided a special micro-environment for AuNPs’ growth. Pyknotic AuNPs were modified onto the cellulose by an in situ growth technique and they were distributed onto the fibers’ surface uniformly (Figure 2B). Moreover, the conductivity of the paper-based electrode was measured by a four-probe tester (Agilent U1251B multimeter), with the results shown in Figure S1. Significantly, PWE displayed lower resistance (0.385 Ω) than that of new clean FTO (2.017 Ω) and ITO (1.337 Ω). Then, deposition of Cu_2_O/CuO on the PWE was conducted by an in situ synthesis approach with two steps. To better survey the preparation process of the Cu_2_O, samples were gathered at diverse stages of photochemical reaction time. At first, layered substances (Figure S2A) with a particle structure were clearly observed adsorbing on the surface of the PWE. After 15 min irradiation, a small amount of crystals similar to microcubes were sparsely distributed on the fibers (Figure S2B). To additionally probe the existing elements, energy-dispersive X-ray spectroscopy (EDS) and element mapping analysis were performed. Clearly, it could be seen that peaks of tungsten are observed in the EDS spectra (Figure S3) for working electrodes and Figure S2B_1_–B_4_ reveals that Cu, W, O, and C elements were uniformly distributed on the fibers. Hence, layered substances were presumed to be CuWO_4_. In addition, with the increase of illumination time, the distribution of cubes became more and more intensive until it reached optimum at the photoreaction time of 60 min (Figure S2C,D, and Figure 2D). Only Cu, O, and C peaks were collected for PWE/Cu_2_O (Figure 2C) and they were evenly distributed on the fibers (Figure 2E). The fact that no spectrum pertaining to the tungsten element was found in EDS patterns of electrodes with 60 min photoreaction time demonstrated that CuWO_4_ was gradually converted to oxides of microcubes with the increasement of photoreaction time. The more microscopic morphology of cubes was observed by zoom-in SEM with ~400 nm diameter (inset in Figure 2D). Moreover, there was a sparse distribution of cubes on the PWE (Figure S2E) without the photoreaction auxiliary unit for photochemical processes, verifying that the existence of the photoreaction auxiliary unit was necessary. The separate working electrode only has a growth fluid capacity of 50 μL, whereas the electrode with the photoreaction auxiliary unit could hold up to 1.0 mL. Moreover, the SEM image of the working electrode without illumination showed unsatisfactory performance (Figure S2F), illustrating the necessity of illumination. In the subsequent reaction process under alkaline condition, Cu_2_O in cubes was gradually partly transformed into intersecting sheet CuO (Figure 2F).

**Figure 3:**
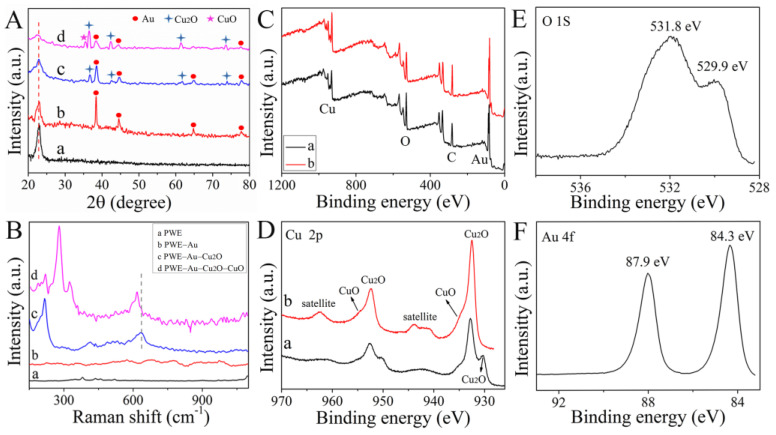
(**A**) XRD patterns and (**B**) Raman spectra (a) cellulose paper, (b) PWE, (c) PWE/Cu_2_O, and (d) PWE/Cu_2_O/CuO. (**C**) XPS survey scan of (a) PWE/Cu_2_O and (b) PWE/Cu_2_O/CuO. **(D**) High−resolution XPS spectra of Cu 2p of (a) PWE/Cu_2_O and (b) PWE/Cu_2_O/CuO. High−resolution XPS spectra of (**E)** O 1S and (**F**) Au 4f of PWE/Cu_2_O/CuO.

The phase structure and the crystallization details of the sample were further researched with X-ray diffraction (XRD) and Raman spectroscopy. A peak at 22.89° gathered from the original working zone of the paper-based device was assigned to (002) planes of cellulose [31], as depicted in Figure 3A. For PWE, four additional peaks at 38.18, 44.39, 64.58, and 77.55° were well-matched with characteristic peaks of Au NPs (JCPDS 04-0784). Except for naked papers and Au NPs peaks, other distinguishable peaks in curve c could be well-indexed to the pristine cubic phase of Cu_2_O shown in Figure S4A. The PWE/Cu_2_O/CuO electrode exhibited apparent diffractions of cubic Cu_2_O and additional diffraction peaks at 35.49° of CuO, illustrating successful preparation of Cu_2_O/CuO. Furthermore, Raman spectra of above electrodes were shown in Figure 3B. Similar to that of cellulose paper, no obvious characteristic peaks were recorded in the Raman pattern of PWE. After the first photoreaction, three peaks were collected for the Cu_2_O-functionalized PWE. Peaks at 217 and 636 cm^−1^ resulted from the second-order Raman-allowed mode and the red-allowed mode of Cu_2_O, meanwhile another presented at 413 cm^−1^ corresponding to the four-phonon mode. Both Cu_2_O and CuO peaks were recorded simultaneously for PWE/Cu_2_O/CuO. Apart from the peak of Cu_2_O at 217 cm^−1^, the other three peaks at 280, 326, and 617 cm^−1^ were assigned to Ag, Bg, and Bg mode of CuO. All these peaks exhibited sharpness, indicating preferable crystallization of the synthesized materials.

X-ray photoelectron spectroscopy experiments were further operated to gain chemical composition information of the PWE/Cu_2_O and PWE/Cu_2_O/CuO. As shown in Figure 3C, the presence of Cu, O, and Au elements could be attributed to the product of the photochemical reaction and conductive Au NPs. To explore the chemical state of the Cu element, the Cu 2p core-level spectra of PWE/Cu_2_O and PWE/Cu_2_O/CuO were compared as displayed in Figure 3D. For Cu_2_O-modified PWE, the characteristic peaks at the binding energies of 932.8 and 952.6 eV were associated with the Cu 2p3/2 and Cu 2p1/2 of Cu_2_O, respectively [32]. As a contrast, PWE/Cu_2_O/CuO showed two additional obvious peaks at 934.8 and 954.6 eV, which corresponded to Cu 2p3/2 and Cu 2p1/2 of CuO. Additionally, three weak satellite peaks at 940.8, 943.8, and 962.3 eV originated from multiple excitations in copper oxides and were attributed to the open 3d^9^ shell of Cu^2+^ in CuO [33]. As shown in Figure 3E, the peak at 529.9 eV was related to the lattice oxygen from both Cu_2_O and CuO phases and the other peak at 531.8 eV marked the surface hydroxyl group, which was consistent with the reported literature [34]. Moreover, Au 4f 7/2 and Au 4f 5/2 peaks presented at 84.3 and 87.9 eV were observed (Figure 3F), which were similar to the counterpart for the standard Au (0) [35]. Moreover, the solution’s color change in the whole process was also favorable evidence for the successful preparation of Cu_2_O/CuO (Figure S4B). All of these results matched each other, demonstrating the successful fabrication of PWE/Cu_2_O/CuO.

### 3.2. Ce-Based Materials Characterization

**Figure 4:**
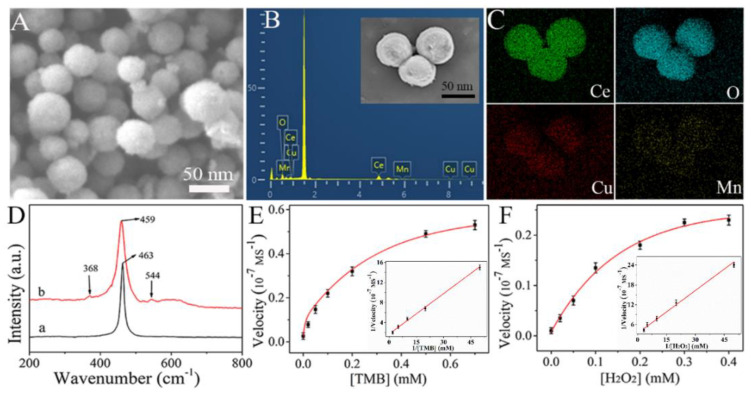
(**A**) SEM image, (**B**) EDS, and (**C**) mapping of CuMn@CeO_2_. (**D**) Raman spectra of (a) CeO_2_ and (b) CuMn@CeO_2_. Michaelis–Menten curves at (**E**) fixed concentration of H_2_O_2_ (2 mM) and various concentrations of TMB (0–0.7 mM) and (**F**) fixed concentration of TMB (0.6 mM) and various concentrations of H_2_O_2_ (0−0.4 mM) (inset: corresponding Lineweaver−Burk plots).

The SEM technique was characterized to explore the morphology of the beacon in colorimetric reactions. Obviously, uniform-size nanospheres with a rough surface were found and the average size of CuMn@CeO_2_ was around 60 nm (Figure 4A). To probe the elements, EDS and mapping were conducted, with the results displayed in Figure 4B,C, respectively. It could be observed that Ce, Cu, Mn, and O elements were collected and they were evenly distributed in the nanospheres. Furthermore, the main peak of CeO_2_ in Raman spectra at about 463 cm^−1^ was attributed to the F_2g_ Raman mode (Figure 4D). Compared with that of pure CeO_2_, the peak shifts to lower frequency at around 459 cm^−1^, indicating occurrence of defects in the CeO_2_ lattice structure after doping with Cu and Mn metals [36]. Meanwhile, additional peaks at 368 and 544 cm^−1^ were collected, which correspond to oxygen vacancies for CuMn@CeO_2_, confirming that extra oxygen vacancies existed in CuMn@CeO_2_ with excellent catalytic performance.

Additionally, steady-state kinetic constants measurement experiments were carried out to verify catalytic performances of CuMn@CeO_2_ (Figure 4E,F). During the process, TMB and H_2_O_2_ concentration were variables. Obtained data were fitted by the Michaelis–Menten kinetic equation, v = (v_max_ × [S])/(Km + [S]), where a smaller Km value means a higher affinity of enzyme to substrate. Compared with that of horseradish peroxidase (Km(H_2_O_2_) = 3.7 mM, Km(TMB) = 0.434 mM), the values for CuMn@CeO_2_ towards H_2_O_2_ and TMB were 0.15 mM and 0.12 mM, indicating that CuMn@CeO_2_ had a stronger affinity for H_2_O_2_ and TMB than enzyme and provided accessible active sites for analytes.

### 3.3. EIS and PEC Behavior

**Figure 5:**
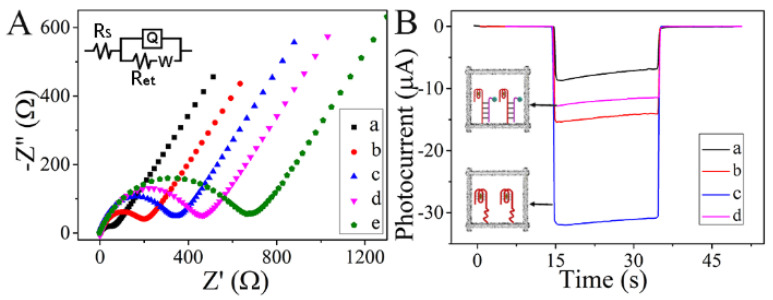
(**A**) EIS spectra of (a) PWE, (b) PWE/Cu_2_O, (c) PWE/Cu_2_O/CuO, (d) PWE/Cu_2_O/CuO/hemin−DNA_1_, and (e) PWE/Cu_2_O/CuO/hemin−DNA_1_/CuMn@CeO_2_−DNA_2_ in 5 mM [Fe(CN)_6_]^3−/4−^ solution containing 0.1 M KCl. (**B**) Photocurrent responses of (a) PWE/Cu_2_O, (b) PWE/Cu_2_O/CuO, (c) PWE/Cu_2_O/CuO/hemin−DNA_1_, and (d) PWE/Cu_2_O/CuO/hemin−DNA_1_/CuMn@CeO_2_−DNA_2_ in 0.01 M PBS solution.

Electrochemical impedance spectroscopy (EIS) was an effective technique to verify the manufacturing procedure of the sensing platform [37]. As magnified in Figure 5A, PWE showed a small electron-transfer resistance (*R_et_*) due to great conductivity of AuNPs (curve a). With the nanomaterial modification progress, the *Ret* increased dramatically for PWE/Cu_2_O and PWE/Cu_2_O/CuO (curves b and c) due to low conductivity of Cu_2_O and CuO. As expected, the *R_et_* showed an upward trend when hemin-DNA_1_ and CuMn@CeO_2_-DNA_2_ were attached onto the PWE surface in turn (curves d and e). It could be explained as the steric hindrance of hemin-DNA_1_ and CuMn@CeO_2_-DNA_2_ that prevented the charge-transfer rate of redox. Furthermore, the transient photocurrent responses of modified PWE were monitored. As revealed in Figure 5B, PWE/Cu_2_O/CuO (curve b) displayed an obvious enhancement of PEC recovery compared with PWE/Cu_2_O (curve a) due to the heterojunction formation of Cu_2_O/CuO. Significantly, PEC performance of a paper-based electrode with greater immobilization capacity for photoelectric materials caused by special three-dimensional interlaced fibers was superior to FTO/ITO-based electrodes (Figure S5). As the electrode incubated with hemin-DNA_1_, the value of photocurrent increased dramatically due to the presence of G-quadruplex/hemin (curve c). With the addition of CuMn@CeO_2_-DNA_2_, a reduced photocurrent was acquired (curve d) caused by enhanced steric hindrance and reduced electron transfer efficiency, indicating that the biosensor was successfully prepared as expected.

### 3.4. Analytical Performance

**Figure 6:**
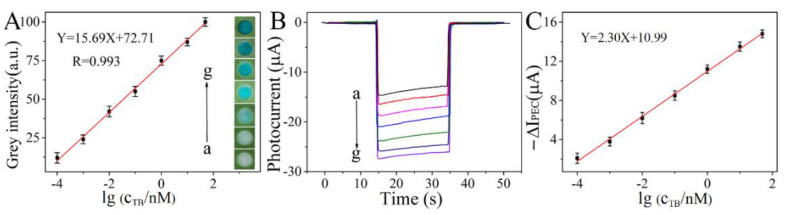
(**A**) Calibration curve between color intensity and logarithm of TB concentration (inset: color intensity). (**B**) Photocurrent of sensing device at different TB concentrations (0.0001, 0.001, 0.01, 0.1, 1, 10, 50 nM) and (**C**) corresponding calibration curve between −ΔI_PEC_ and logarithm of TB concentration.

The well-designed signal output paper-based device was employed to realize the quantitative detection by analyzing the PEC signals and color intensity with the variety of TB concentrations. Excellent catalytic performances toward H_2_O_2_ of CuMn@CeO_2_ can offer visual prediction for analytes. Under optimal conditions (Figure S6), color intensity and photocurrent witnessed an upward trend along with the elevated concentration of TB, and a dynamic range was obtained from 0.0001 to 50 nM (Figure 6A,B). Meanwhile, the excellent linear relationship between logarithmic value of TB concentrations (Figure 6C) and photocurrent response could be fitted and the linear regression was −ΔI_PEC_ (µA) = 2.30 lgc + 10.99 (R = 0.994), and the detection limit was calculated as 0.035 pM (S/N = 3). Compared with single-signal readout strategies (Table 1), the paper-based visual/PEC biosensor exhibited apparent merits in wide response range and dual-signal sensing mode, providing a more accurate and effective method for TB detection.

### 3.5. Specificity, Stability, and Reproducibility

**Figure 7:**
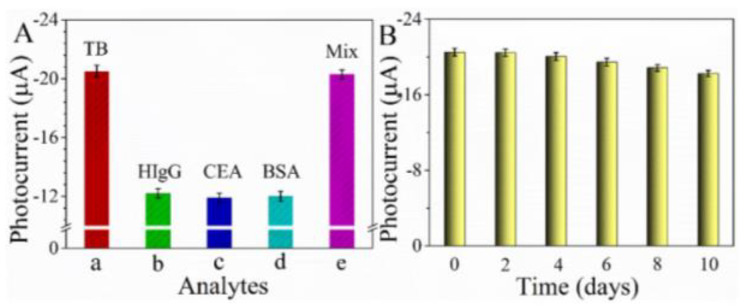
(**A**) Photocurrent of the bioassay in the presence of 0.1 nM sample. (**B**) Photocurrent responses of constructed PEC sensing platform with 0.1 nM TB.

To further assess the feasibility of the present protocol, three significant criterions for biosensors, the selectivity, stability, and reproducibility, were explored. The specificity of the dual-signal bioassay was investigated by incubation with 0.1 nM TB, human immunoglobulin G (HIgG), carcinoembryonic antigen (CEA), bovine serum albumin (BSA), and their mixture. The highest photocurrent response was obtained for samples containing TB (Figure 7A), demonstrating gratifying specificity. The stability of the as-prepared sensing device was measured by applying 0.1 nM TB as samples and monitoring the photocurrent responses intermittently (every 2 days). As expected, experimental results revealed acceptable stability (Figure 7B). Furthermore, the reproducibility was investigated by testing five independent electrodes, and the relative standard deviation of photocurrent response was 4.3%, demonstrating acceptable precision and repeatability.

## 4. Conclusions

Herein, Cu/Mn-doped CeO_2_ and branched sheet embedded Cu-based nanocubes were prepared for fabrication of an effective visual/PEC paper-based sensing platform to obtain sensitive analysis of TB. The rapid chromogenic reactions were achieved by the release of CuMn@CeO_2_ with the addition of TB, which provides a simple visual prediction. Meanwhile, the presence of TB led to dissociation of CuMn@CeO_2_ and the electrons-acceptable distance of G-quadruplex/hemin, which then contributed to the switchover of the original PEC signal caused by branched sheet embedded nanocubic Cu_2_O/CuO. Undoubtedly, such dual-signal output strategy with good performance was capable of making detection results more sensitive and accurate. Additionally, it was anticipated that the approach shows potentiality in designing numerous biosensors with excellent performance for analytes detection.

## Figures and Tables

**Table 1:** Comparison of the biosensor with other analytical strategies.

Method	Materials	Liner Range (nM)	Detection Limit (pM)	References
Electrochemiluminescence	Graphene oxide and carbon nanotubes	0.001–5	0.23	[38]
Electrochemical	Au NPs	0.005–50	1.1	[39]
Electrochemical	Au electrode and methylene blue	0.005–1	1.7	[40]
Fluorescence	Zinc selenide quantum dots	0.1–20	25	[41]
PEC	Au-ZnO Nanoflowers	0.001–30	0.37	[7]
Visual/PEC	Cu_2_O/CuO and CuMn@CeO_2_	0.0001–50	0.035	This work

## Data Availability

Not applicable.

## Supplementary Materials

The following supporting information can be downloaded at: https://www.mdpi.com/article/10.3390/bios13020227/s1, Figure S1: Physical photos of (A) FTO, (B) ITO, and (C) PWE sheet resistance; Figure S2: SEM images of PWE with photoreaction progresses at (A) 0 min, (C) 30 min, (D) 45 min. (B–B4) Elemental mappings of PWE with photoreaction progresses at 15 min. SEM images of (E) PWE/Cu_2_O without photoreaction auxiliary unit and (F) the electrode without illumination; Figure S3: EDS spectra of PWE with photoreaction progresses at 15 min; Figure S4: (A) XRD pattern of Cu_2_O and CuO. (B) Photographs of photoreaction progresses at (a) 0 min, (b) 15 min, (c) 30 min, (d) 45 min and (e) 60 min; and (f) the obtained Cu_2_O/CuO photograph; Figure S5: Photocurrent response of (a) FTO/Cu_2_O/CuO, (b) ITO/Cu_2_O/CuO, and (c) PWE/Cu_2_O/CuO; Figure S6: Effect of TB incubation time. References [42,43] are cited in the Supplementary Materials.
