# Supplementary material for: Visual/Photoelectrochemical Off-On Sensor Based on Cu/Mn Double-Doped CeO2 and Branched Sheet Embedded Cu2O/CuO Nanocubes
Source: Biosensors (Basel). 2023 Feb 4;13(2):227. doi: 10.3390/bios13020227 (PMC9954256; doi:10.3390/bios13020227)
Supplement: Supplementary file 1 [file biosensors-13-00227-s001.zip › biosensors-2084823-supplementary.pdf]

Supplementary Material

Visual/Photoelectrochemical off-on Sensor Based on Cu/Mn Double-doped CeO_2 and Branched Sheet Embedded Cu_2O/CuO Nanocubes

Huihui Shi[1], Yanfei Che[1], Yumeng Rong[1], Jiajun Wang[1], Yanhu Wang[2], Jinghua Yu[1] and Yan Zhang[1,3,*]

[1]*School of Chemistry and Chemical Engineering, University of Jinan, Jinan 250022, China*

[2]*Shandong Analysis and Test Center, Qilu University of Technology (Shandong Academy of Sciences), Jinan 250014, China*

[3]*Key Laboratory of Optic-electric Sensing and Analytical Chemistry for Life Science, MOE, Qingdao University of Science and Technology, Qingdao 266042, China*

*Correspondence: chm_zhangyan@hotmail.com

1. Experimental Section

1.1 Reagents

All reagents are of analytical reagent grade and directly used for the experiments. Ultrapure water was obtained from a Lichun water purification system (resistivity≥18.25 MΩ cm). The chromatographic paper was purchased from GE Healthcare Worldwide and used with further regulation to A4 size. Chloroauric acid, cerium nitrate hexahydrate ($Ce(NO_3)_3{\cdot}6H_2O$, copper chloride ($CuCl_2{\cdot}2H_2O$), manganese chloride ($MnCl_2$), polyvinylpyrrolidone (PVP), and ethylene glycol (EG) were purchased from Sinopharm Chemical Reagent Co. Ltd. (Shanghai, China). 3-Aminopropyltriethoxysilane (APTES) was obtained from J&K Scientific Ltd. (Beijing, China). Hydroxylamine hydrochloride, glucose, sodium citrate, sodium hydroxide and copper nitrate hexahydrate ($Cu(NO_3)_2{\cdot}6H_2O$) were ordered from Sinopharm Chemical Reagent Co., Ltd. (Shanghai, China). Sodium tungstate (Na_2WO_4) was supplied by Macklin Reagent Co., Ltd. (Shanghai, China). The DNA sequences are given below.

DNA_1: TTTTTTTTTTTTCCAACCACACCAACCAAGGGTTGGGCGGGATGGTT

DNA_2: TTTTTGGTTGGTGTGGTTGG

1.2 Apparatus

Scanning electron microscopy (SEM) images were obtained using a QUANTA FEG 250 thermal field emission scanning electron microscopy (FEI Co., USA). X-ray diffraction (XRD) patterns were collected on a D8 advance diffractometer system equipped with Cu Ka radiation (Bruker Co., Germany). Raman spectra were measured by micro-Raman spectrometer (Renishaw INVIA Reflex). X-ray photoelectron spectroscopy (XPS) measurements were achieved with an ESCALAB 250Xi photoelectron spectrometer. Electrochemical impedance spectroscopy (EIS) were performed using an IM6x electrochemical work station (Zahner Co., Germany). And the photocurrent were measured on a CHI 660D electrochemical workstation (Shanghai Chenhua Instruments Corporation, China) with a three-electrode system.

1.3 Preparation of $CuWO_4$

0.2 mol $Cu(NO_3)_2\cdot 6H_2O$ was dissolved in deionized water and precipitated by slow addition of 0.2 mol Na_2WO_4 aqueous solution under continuous stirring. The precipitate was washed with deionized water and dried in an oven at 75 °C for 5 h. Finally, the collected product was calcined at 500 °C for 5 h to obtain $CuWO_4$ as precursor.

1.4 Preparation of PWE

Au NPs were coated on cellulose paper by in situ growth approach where Au (III) was reduced to metallic gold with the catalysts of Au NP seeds [1]. Briefly, 50 μL of as-prepared Au NP seeds solution was transferred to working zone followed by placing for 1 h to optimize the immobilization of Au NP seeds on cellulose fibers. After rinsing with ultrapure water for twice, growth aqueous solution (50 μL) containing 1% chloroauric acid (25 μL) and 0.1 M hydroxylamine hydrochloride was applied to working electrode. Ultimately, the electrode was rinsed to remove loosely bound Au NPs after incubated for 1 h at room temperature.

1.5 Preparation of CuMn@CeO_2

CuMn@CeO_2 was prepared according to previous report with slight modification [2]. Briefly, 0.5 g $Ce(NO_3)_3\cdot 6H_2O$ and 0.2 g PVP were dissolved in 30 mL EG with stir and kept for 30 min. Then 0.02 g $CuCl_2\cdot 2H_2O$ and 0.02 g $MnCl_2$ were added and the above solution was transferred into Teflon-lined autoclave and heated for 8 h at 180 °C. After precipitate was collected and washed thoroughly with water and ethanol, the prepared products dried at 60 °C for 12 h and finally calcined at 450 °C.

2. Results and Discussions

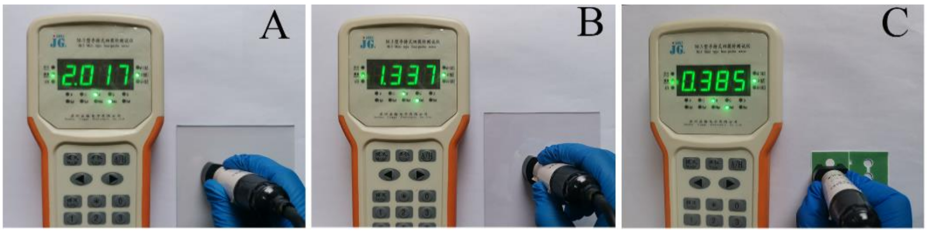

Figure S1 Physical photos of (A) FTO, (B) ITO, and (C) PWE sheet resistance.

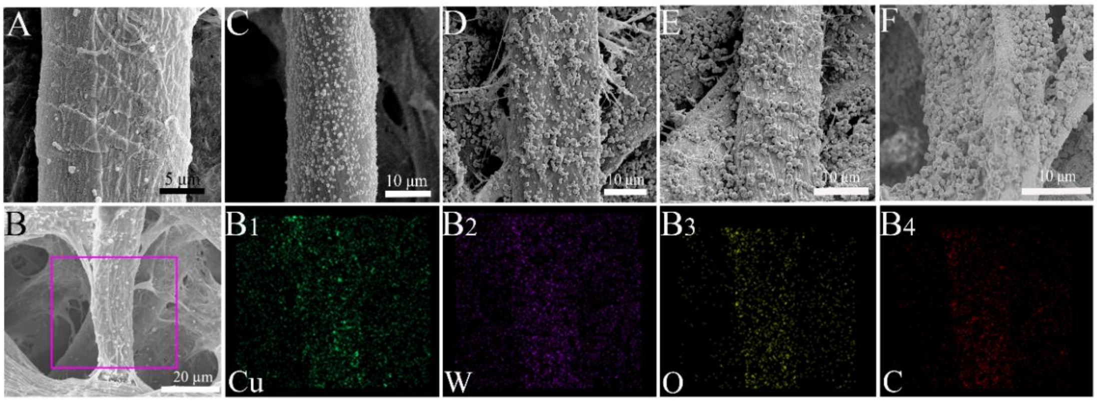

Figure S2 SEM images of PWE with photoreaction progresses at (A) 0 min, (C) 30 min, (D) 45 min. (B-B_4) Elemental mappings of PWE with photoreaction progresses at 15 min. SEM images of (E) PWE/Cu_2O without photoreaction auxiliary unit and (F) the electrode without illumination.

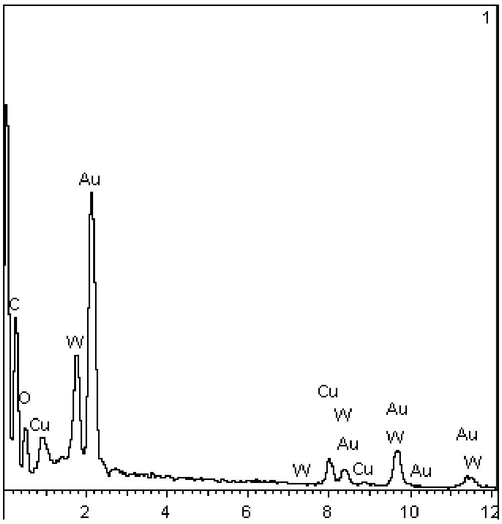

Figure S3 EDS spectra of PWE with photoreaction progresses at 15 min.

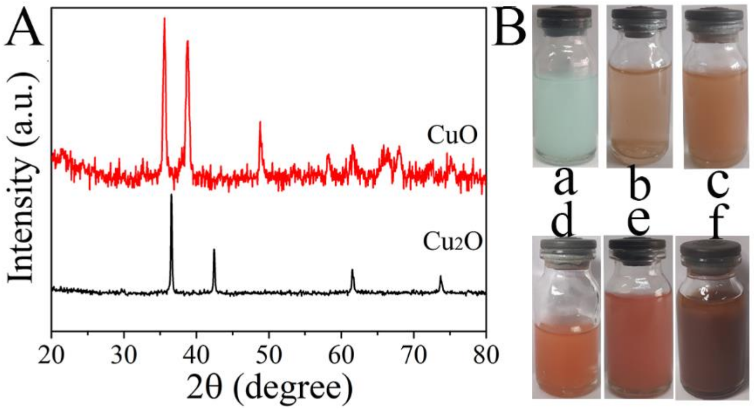

Figure S4 (A) XRD pattern of Cu_2O and CuO. (B) Photographs of photoreaction progresses at (a) 0 min, (b) 15 min, (c) 30 min, (d) 45 min and (e) 60 min; and (f) the obtained Cu_2O/CuO photograph.

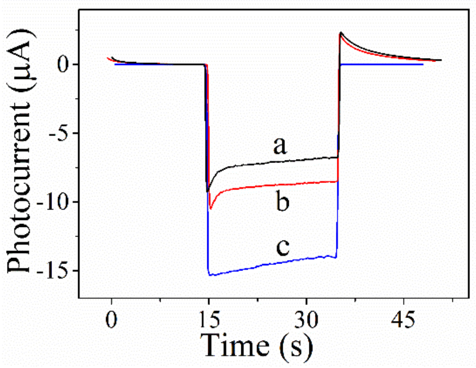

Figure S5 Photocurrent response of (a) FTO/Cu_2O/CuO, (b) ITO/Cu_2O/CuO, and (c) PWE/Cu_2O/CuO.

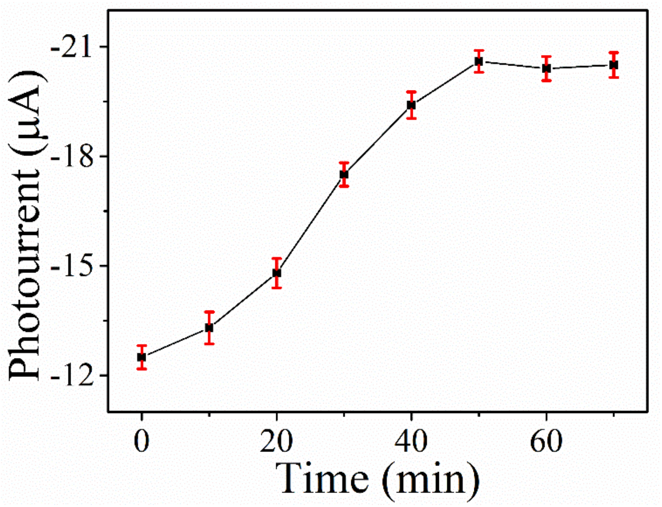

Figure S6 Effect of TB incubation time.

References

1. Wang H.; Jian Y.; Kong Q.; Liu H.; Lan F.; Liang L.; Ge S.; Yu J. Ultrasensitive electrochemical paper-based biosensor for microRNA via strand displacement reaction and metal-organic frameworks. *Sens. Actuators B: Chem.* **2018**, 257, 561-569.
2. Li X.; Zhang H.; Tang Y.; Wu P.; Xu S.; Zhang X. A both-end blocked peroxidase-mimicking DNAzyme for low-background chemiluminescent sensing of miRNA. *ACS sensors* **2017**, 2, 810-816.
